# The truth about co-benefits: a multidimensional feasibility assessment for thailand and beyond

**DOI:** 10.1088/2515-7620/adae5e

**Published:** 2025-02-07

**Authors:** Eric Zusman, Kaoru Akahoshi, Tatsuya Hanaoka, Christopher S Malley, Supat Wangwongwatana, Nutthajit Onmek, Ittipol Paw-armart, Kim Oanh Nguyen Thi, Lai Nguyen Huy, Johan C I Kuylenstierna, Tomoki Hirayama, Yurie Goto, Kawashima Kazumasa, Markus Amann, Zbigniew Klimont, Jessica Slater

**Affiliations:** 1 The Institute for Global Environmental Strategies, Japan; 2 National Institute Environmental Studies, Japan; 3 Stockholm Environment Institute, University of York, York, United Kingdom; 4 Thamasatt University, Thailand; 5 Bansomdejchaopraya Rajabhat University, Thailand; 6 Pollution Control Department, Thailand; 7 Environmental Engineering and Management, Asian Institute of Technology, Pathumthani, Thailand; 8 Center for Nexus of Air Quality, Health, Ecosystem and Climate, Asian Institute of Technology, Pathumthani, Thailand; 9 Mizuho Research and Technologies, Japan; 10 Mitsubishi UFJ Research and Consulting, Japan; 11 World Bank, Washington D.C., United States of America; 12 International Institute for Applied Systems Analysis, Laxenburg, Austria

**Keywords:** co-benefits, integrated assessment models, multidimensional feasibility, barrier analysis, accelerating transitions

## Abstract

Research has yielded increasingly robust estimates of the co-benefits from mitigating climate change while reducing air pollution, improving health, and meeting other development needs. Though quantifying these often hidden benefits could ease cost concerns and lower technological constraints for development-friendly climate solutions, achieving co-benefits frequently requires overcoming difficult-to-measure social and institutional barriers. This study extends insights from research focusing on quantitatively assessing the feasibility of a 1.5 °C future to build a multidimensional framework for measuring different barriers to achieving co-benefits. The framework offers a novel yet generalizable approach for bringing context-appropriate assessments of different dimensions of feasibility into the integrated assessment modelling that underpins work on co-benefits. It then outlines five steps for applying that framework to evaluate the size of different barriers for transport, agricultural and residential energy co-benefit solutions in Thailand. The results demonstrate that the sum of the delays from social/institutional barriers exceed economic/technological barriers for four out of six studied solutions. These delays also lead to increases of 24% to 31% in PM_2.5_ emissions relative to a no-barriers effective implementation scenario between 2015 and 2030 and 2040. The feasibility framework can be integrated into not only national policy scenarios but also project assessments, following trends in carbon finance. An international barriers database as well as strengthening links to work on barriers and technological diffusion, transaction costs, and multi-level transitions can also help spread multi-dimensional feasibility assessments across countries and scales.

## 1. Introduction

In recent years, policymakers have realized that the climate crisis is less a possibility than an inevitability. This realization has led to a growing interest in transitions aimed at limiting warming below 1.5 °C compared to pre-industrial levels (IPCC 2018 2022).^13^^13^The IPCC defines transitions as the ‘the process of changing from one state or condition to another in a given period of time [for] individuals, firms, cities, regions and nations, and can be based on incremental or transformative change.’ In this article, transitions will apply chiefly to changes at the national level and the changes that countries make to their policies and enabling environments that would help achieve the 1.5 °C targets under the Paris Agreement. The sociotechnical changes that can support these transitions have also drawn growing interest. Many changes in the transport, agricultural and residential sectors featuring in these transitions can mitigate greenhouse gas (GHGs) as well as air pollutants. Some of those air pollutants are also short-lived climate pollutants (SLCPs) (Bond *et al* 2013, Shindell *et al* 2017).^14^^14^Recent research has suggested that the impacts of black carbon on warming are lower than initially anticipated (Takemura 2020). However, the impacts on regional climate systems through, for instance, changes in precipitation patterns are still envisaged as being significant (Ramanathan *et al* 2005). By helping to control multiple pollutants, these options can deliver co-benefits and contribute to the Sustainable Development Goals (SDGs). For example, many of the relevant projects and policies in this article reduce air pollution, cut seven million early deaths annually, and avoid millions of tons of crop damages and associated economic losses (UNEP/WMO 2011a, WHO 2014, Haines *et al* 2017, Hoffmann *et al* 2021).

Underlying much of the research on co-benefits is a key point: co-benefits can offset mitigation costs and encourage policymakers to invest in climate projects and policies (Krupnick *et al* 2000, Pearce 2000, Uchida and Zusman 2008, Farzaneh *et al* 2021). One might infer from this key point that policymakers would be inclined to implement options with multiple benefits when formulating transition pathways. In fact, several high-profile reports have underlined the potential for co-benefits from measures that curb emissions from vehicles, cookstoves, and burning of biomass (UNEP/WMO 2011b, UNEP APCAP and CCAC 2019). These reports rely heavily on integrated assessment models to identify how much technological and social changes contribute to climate and other development goals.

While the above models are critical to assessing the size of benefits, they have limitations (McMichael 1997, Schneider 1997, Norgaard and Baer 2005, Ackerman *et al* 2009, Mathias *et al* 2020). An important limitation is integrated assessment models do not explicitly consider the feasibility of implementing recommended solutions (Nielsen *et al* 2020, Brutschin *et al* 2021, Hickmann *et al* 2022).^15^^15^The IPCC defines ‘feasibility’ as ‘the potential for a mitigation or adaptation option to be implemented [and notes that feasibility] depends on geophysical, environmental-ecological, technological, economic, sociocultural and institutional factors that enable or constrain the implementation of an option.’ (E1. Footnote 72). The inattention to social and institutional feasibility may be particularly problematic because effective implementation of co- benefit interventions often requires social and institutional enabling reforms (UNEP/WMO 2011, UNEP APCAP and CCAC 2019). To some degree, the limited attention to these issues is understandable: the difficulties measuring the effects of administrative capacity/coordination (i.e. institutional dimensions) or awareness/user acceptability (i.e. social dimensions) make their integration into modeling difficult (Nielsen *et al* 2020, Brutschin *et al* 2021, Hickmann *et al* 2022). Yet, the failure to include these constraints into models may generate false hope about achieving possible results (Li, 2017). The exclusion of these consideration may also weaken recommendations for enabling reforms^16^^16^The IPCC suggests that ‘‘enabling conditions’ refers to conditions that enhance the feasibility of adaptation and mitigation options. Enabling conditions include finance, technological innovation, strengthening policy instruments, institutional capacity, multi-level governance and changes in human behavior and lifestyles’ (E1. Footnote 73). such as the interagency coordination and capacity building mechanisms required to implement solutions at scale.

The purpose of this study is to demonstrate a novel approach for incorporating feasibility into co-benefits modeling. To illustrate that approach, the article uses original survey data from Thailand on barriers to implementing solutions from the transport, agricultural and residential energy sectors. The study shows that the institutional and social constraints could often slow the implementation more than economic and technological constraints. A policy-level application of the findings suggests the delays result in increases of 24% to 31% in PM_2.5_ emissions relative to a no-barriers effective implementation scenario in Thailand between 2015 and 2030 and 2040 respectively. The feasibility framework can also be used to analyze barriers at smaller scales—for example, carbon finance projects.

The study is divided into five sections. The second section reviews literature on co-benefits and feasibility. The third section outlines an approach for bringing feasibility into modeling frameworks with the case of Thailand. The fourth section discusses the implications of this approach for research and policy. The final section reiterates conclusions and highlights areas for future research.

## 2. Literature review on co-benefits and feasibility

This article brings together literature on co-benefits and feasibility. Research on co-benefits traces back more than three decades (Schneider 1989, Ayres and Walter 1991, Glomsrød *et al* 1992, Pearce 1992, Elkins 1996). The earliest first wave of studies on co-benefits demonstrated that climate policies such as a carbon tax could limit air pollution and bring significant health benefits in *developed* countries (Elkins 1996, Pearce 2000). The health benefits were often large enough to offset mitigation costs—for instance, air quality co-benefits could be more than ten times larger than climate benefits in some developed country cases (Pearce 1992). These savings could conceivably motivate policymakers to support reforms needed to achieve the estimated gains (Pearce 1992, Krupnick *et al* 2000, Pearce 2000).

A second wave of co-benefits research looked at a broader range of sectoral policies and projects in *developing countries* (Bussolo and O’Connor 2001, Aunan *et al* 2004, Morgenstern *et al* 2004). These studies often found greater potential savings for a more diverse set of interventions (Li and Crawford-Brown 2011, Menikpura *et al* 2013, Challcharoenwattana and Pharino 2015, Dhar and Shukla 2015, Jiang *et al* 2016, Pathak and Shukla 2016, Dhar *et al* 2017, Li *et al* 2018, Liu *et al* 2018, Shi *et al* 2022). The larger size of benefits was due to poorer air quality and higher population densities in developing countries (Nemet *et al* 2010). Therefore, efforts to mitigate climate change would also lead to greater reductions in air pollution for more people (Bollen *et al* 2009). This finding could lower concerns that investing in climate mitigation would divert resources from other priorities in developing countries (Zusman 2008).

Following this second wave of developing country-focused research, some of a third wave of work on co-benefits has concentrated on influencing policy (Cai *et al* 2023, Karlsson *et al* 2023, Roggero, Gotgelf, and Eisenack 2023). To illustrate, several overview studies have mapped connections between energy policies and streams of different benefits to help increase impacts on policy (Karlsson *et al* 2020). To offer another example of the policy-orientation, many studies have focused on solutions that curb air pollutants known as SLCPs such as black carbon, tropospheric ozone, and methane (UNEP/WMO 2011b, Bond *et al* 2013, Shindell *et al* 2017).^17^^17^Note that IPCC Joint 1st and 2nd IPCC Expert Meeting on Short-lived Climate Forcers (SLCFs) recommends using ‘SLCP’ for warming species of air pollutants. For species that have both a warming and cooling effect, it calls for using ‘SLCFs.’


A related trend is an interest in how to not only change policy but trigger action on the ground. To illustrate, with the support of international and regional initiatives such as the Climate and Clean Air Coalition (CCAC) and Asia Pacific Clean Air Partnership (APCAP), high-profile reports have identified 25 action-oriented clean air solutions that could improve air quality and mitigate near- and long-term climate change in Asia (UNEP APCAP and CCAC 2019). Similar reports have been developed for Latin America and Africa (CCAC and UNEP 2016, Hannah *et al* 2021) as well as countries such as Bangladesh and Cambodia (Kuylenstierna *et al* 2020, Malley *et al* 2022); a sub-regional report for Southeast Asia is near publication (CCAC 2023).

While this third wave of research on co-benefits has become more policy-relevant and action-oriented, it has still placed a greater emphasis on modelling potential benefits than assessing barriers to achieving them. The inattention to implementation is arguably a function of the fact that much of the work on co-benefits relies on methods that do not integrate feasibility into their analyses. Some studies have acknowledged these shortcomings (Ürge-Vorsatz *et al* 2014, Mayrhofer and Gupta 2015). Others have made modest attempts to overcome them by emphasizing implementation barriers in specific countries (Dubash *et al* 2013) and sectors (Brown et al 2008).

**Figure 1.**
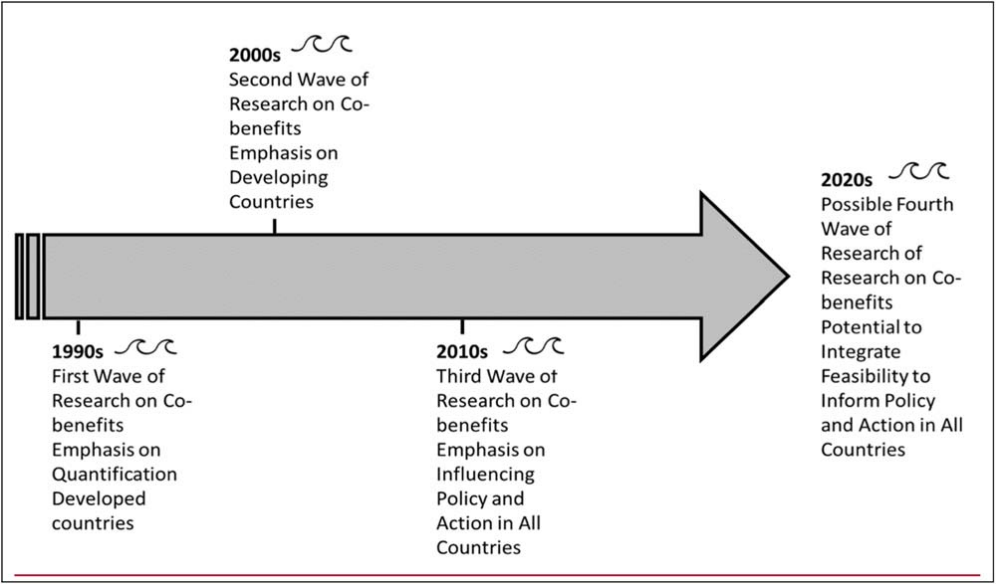
Four waves of co-benefits research.

Another branch of research focusing on feasibility could help bring implementation barriers into a fourth wave of co-benefits research (see figure 1 for an illustration of these waves). Some early work in this space has looked at how different dimensions of feasibility influence whether policies achieve their stated objectives (Majone 1975a, 1975b). Others have taken this insight a step further to use expert surveys and literature reviews to analyze barriers to adopting energy efficiency reforms and renewable energy technologies, paralleling the approach used in this study (Sovacool 2009, Sorrell *et al* 2011, Backlund *et al* 2012). Much of the interest in implementation barriers has considered multiple dimensions of feasibility (Staub-Kaminski *et al* 2014). For instance, some have argued for a ‘2nd best analysis of climate policy’ to give a more realistic assessment how poorly designed policies impact the likelihood of achieving modelling results (Kriegler *et al* 2012).

Yet others have sought to use a similar reasoning to assess the feasibility of 1.5 °C or net zero future (Staub-Kaminski *et al* 2014, Nielsen *et al* 2020, Brutschin *et al* 2021, Hickmann *et al* 2022, Steg *et al* 2022, Ven *et al* 2023). Some studies have underlined that feasibility is critical to close potentially large gaps between integrated assessment models and the real world but acknowledged the difficulties of making the concept sufficiently concrete to integrate into modelling scenarios (Warszawski *et al* 2021). Some have sought close these gaps by using a simple coding technique from an expert literature review for a systematic assessment of different dimensions of feasibility for a range of sectoral mitigation options at the global level (Steg *et al* 2022). Others have shown that there is a need to move beyond sectoral options to demonstrate that social and institutional dimensions of feasibility may limit the likelihood of implementing packages of interventions in broader scenarios at the global and regional levels (Brutschin *et al* 2021). A recent extension of work has used proxy indicators for difficult-to-assess social and institutional constraints (World Bank governance indicators) to empirically estimate how institutional feasibility of realizing reductions in a wide range of modelling scenarios (Bertram *et al* 2024).

The growing attention to feasibility of implementing scenarios in integrated assessment models is welcomed given the urgent need for accelerating 1.5 or net zero transitions. However, none of the recent work on feasibility has focused on creating a generalizable framework that can assess the implementation prospects of national or lower level solutions with co-benefits. The lack of attention is significant because even the more policy and action-oriented work on co-benefits is grounded in the belief that accounting for co-benefits can lower cost concerns and induce technological changes. However, for many of the solutions with the greatest co-benefits, social and institutional barriers may stand in their way of their implementation. There is hence scope to extend work on multiple dimensions feasibility to research on specific co-benefit solutions. The next section describes the case selected to demonstrate how an approach employing expert surveys and literature reviews can integrate feasibility into the co-benefit modelling for a subset of options in Thailand.

## 3. Case selection: transport, residential energy, and open burning solutions in Thailand

To look at feasibility in Thailand, this study focuses on options in the transport, residential energy, and agriculture sectors (table 1). These solutions were selected for three reasons.

**Table 1.** Selected options.

Sector	Option	Brief description
Residential Energy	Replace Traditional Stoves	Adoption of higher efficiency or cleaner stoves, including fan assisted stoves
	Switch to LPG	Switch from solid fuel to liquid petroleum gas powered for cooking
Transport	Promotion of Electric Vehicles (EV)	Adoption and spread of electric vehicles
	Tighter Emission Standards for Vehicles	Introduction of tighter emission standards and energy/fuel efficiency standards for vehicles
	Vehicle Inspection and Maintenance	Vehicle inspection and maintenance that enable early detection and elimination/repair of high emitting vehicles
Agriculture	Control Open Burning and Sustainable Use of Agricultural Residues	Banning or controlling the open burning of agricultural residues, including rice straw, sugarcane and corn

The first is the options are featured in ongoing policies and projects in Thailand. For instance, Thailand adopted a National PM_2.5_ Control Plan in 2019 that includes provisions related to transport (Section 2.1 of the PM_2.5_ Control Plan), open burning (Section 2.3 of the PM_2.5_ Control Plan), and clean cooking (Section 2.5 of the PM_2.5_ Control Plan) (Pollution Control Department 2019). In addition, Thailand is also drafting a Clean Air Law that is likely to aim to curb open burning and vehicle emissions (Walker 2024). Further, to cite a local level example, there are ongoing efforts to acquire carbon finance from voluntary carbon markets for high-efficiency wood burning Kuniokoa cookstoves in rural communities in Thailand. Those efforts, which will be revisited later in the paper, demonstrate the national and international relevance of this intervention (CQuest Capital 2021).

The second reason for selecting these options is they can deliver significant co-benefits (CCAC 2023). For instance, inspection and maintenance programme could lead to sizable reductions in GHGs and air pollutants from the transport sector, including SLCPs. This potential is significant because such programmes target high-emitting (malfunctioning, tampering) vehicles accounting for more than half of the sector’s entire emissions (Hausker 2004, Li 2017). In addition, clean stoves and fuels not only help limiting emissions but can have positive effects on the health of women and children (Rosenthal *et al* 2018). Controls on open burning, meanwhile, can curb pollution and promise more sustainable agricultural yields (Oanh *et al* 2018).

**Figure 2.**
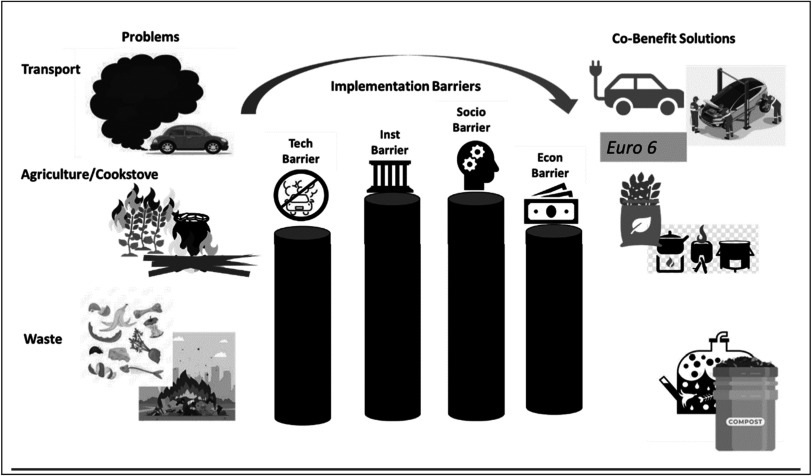
Illustration of problems, barriers and solutions.

The third reason for selecting these options is that achieving their co-benefits is difficult. Several studies have noted that inspection and maintenance of vehicles is challenging, especially in developing countries (Dasgupta *et al* 2001, Hausker 2004, Li and Crawford-Brown 2011, Clean Air Asia 2016, Dandapat *et al* 2020). Similarly, barriers such as low levels of social acceptance, a lack of information, or misplaced government support often stand in the way of clean cookstoves and fuels (Limmeechokchai and Chawana 2007, Chalise *et al* 2018, Thoday *et al* 2018). Efforts to curb open burning of biomass have also easier to demonstrate on paper than achieve in practice (Kanokkanjana and Bridhikitti 2007, Chalise *et al* 2018, Bhuvaneshwari *et al* 2019, Sharma and Jain 2019). In sum, many of the options confront barriers that are not typically quantified in co-benefits modelling (see also figure 2 for an illustration of problems, barriers and solutions).

## 4. Methods

### 4.1. Estimating the size of barriers

One of the keys to estimating how much technological, economic, social, and institutional feasibility affect implementation is quantifying their effects on diffusion. This section describes the estimated size of the barriers belonging to the categories in table 2.

**Table 2.** Defining barriers.

**Technological**	Access to cleaner technology/fuels and technologies/infrastructure enabling implementation.
**Economic**	Costs of cleaner technology fuels as well as policies (i.e. subsidies) that lower prices of resource-intensive options.
**Institutional**	Lack of interagency coordination/capacity as well as design flaws in policies promoting cleaner options.
**Social**	Limited acceptance/awareness of benefits from the clean alternatives as well as a shortage of awareness raising mechanisms/stakeholder engagement mechanisms.

**Figure 3.**
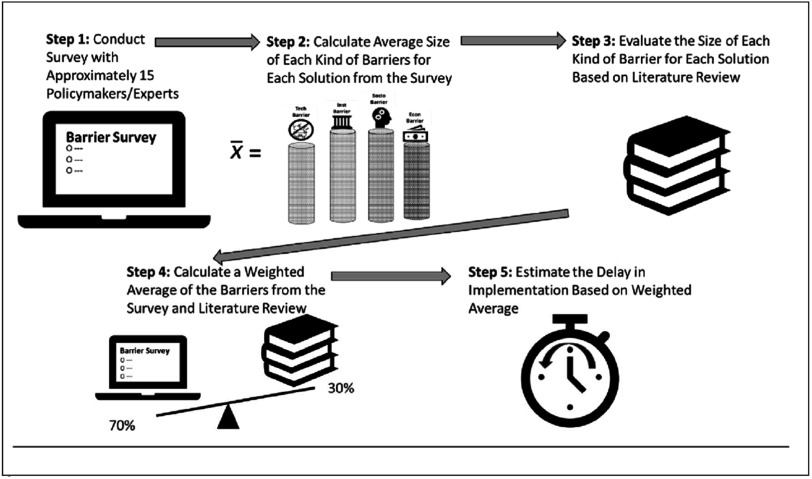
Five step approach to estimating barriers and delays *Source: Authors Diagram*.

To arrive at these estimates, the study went through five steps illustrated in greater detail in figure 3.

First, a barriers survey was distributed to approximately 30 researchers, policymakers and development specialists in mostly Thailand as well as a few additional countries in Southeast Asia. There were three types of surveys: one for transport, one for open burning, and one for residential energy/cookstoves. The respondents were selected based on their deep knowledge of the solution (controls on burning of biomass) or because they worked for a government agency responsible for implementing that solution (regulating mobile source emissions). Most of the respondents were researchers but policymakers accounted for between 10 to 30 percent of the respondents in all three survey areas. The selection of respondents was determined based on consultations with recognized institutions with an extensive network and context-appropriate knowledge of the issue area in Thailand. Survey respondents were told that their titles and positions but not their names would be shared (see appendix for a listing of the backgrounds for each of the survey respondents). The survey asked respondents to assess the size of the influence on the barrier in table 2 on the diffusion of the solutions in table 1; the assumptions in Box 1 were used to code the size of effects.

Box 1. Magnitude of Barriers

The following approach was used to assess the size of barriers for each solution:

• 0% when a response was ‘**no effect’**
• 5% (halfway between 1% to 10%) for **‘small’**
• 15% (halfway between 11% to 20%) for **‘moderate’**

Second, once the approximately 30 responses were received for each type of solution, an average size was calculated for each type of barriers for each solution. For example, an average barrier size was calculated for the technical, economic, social, and institutional barriers for ‘promoting evehicles’ based on the survey responses from the transport survey. See equation (1) for the relevant notation.

\begin{eqnarray*}{\bar{x}}_{k,j}=\displaystyle \frac{\displaystyle {\sum }_{i=1}^{n}{\bar{S}}_{k,j,i}}{n}\end{eqnarray*}

Where

*i* : each expert survey response

*j* : type of barrier (i.e. technological, economic, social and institutional barriers)

*k* : mitigation option shown in table 1

*n* : the total number of responses to expert survey

${\bar{x}}_{k,j}$: average magnitude of barrier type *j* for mitigation option *k* based on the expert survey, which ${\bar{x}}_{k,j}$ ≤ 25%

${\bar{S}}_{k,j,i}$: the size of effects of barrier type *j* for mitigation option *k* from each expert survey response *i*, which ${\bar{S}}_{k,j,i}$ ≤ 25%

Third, the study then complemented the evaluation of the barriers from the surveys with an assessment of relevant literature. The literature review drew from studies concentrating on key sectors and solutions. To the greatest extent possible, the literature was taken from work in Thailand and Southeast Asia (see appendix for list of studies); however, this was not always possible since the research on these solutions in Thailand and Southeast Asia was limited. To translate the literature review into quantitative data, the authors determined if each kind of barrier was ‘small,’ ‘moderate,’ or ‘significant’ for each article based on the criteria presented in table 3. The results of that determination were first entered into table in the appendix to help offer a visual map of the feasibility landscape. The authors then reviewed that landscape to arrive a single ‘no barrier,’ ‘small,’ ‘moderate,’ or ‘significant’ score for each type of barrier for each solution (equation (2)). Note that the final score for literature review assessment was based on the authors’ determination of the overall size of barrier as opposed to a numerical average for the reviewed studies. The decision to use a single overarching score as opposed to an average reflected the difficulties of arriving at an justifiable scheme for weighting studies from different countries, years and disciplines.

\begin{eqnarray*}{x}_{k,j}={S}_{k,j}\end{eqnarray*}

where

**Table 3.** Barrier assessment criteria.

Indicator	Description
No effect	The literature does not mention the barrier.
Small	The literature refers to barriers indirectly and/or briefly though not as a major issue. Moreover, when mentioned, their impact on a solution’s diffusion is limited. For example, in the case of a ‘institutional’ barrier, the literature refers to the need to strengthen implementation capacities, but does not go into depth beyond making this point.
Moderate	The literature refers to the barrier directly, but the impact seems modest. For example, in the case of a ‘social’ barrier, the literature mentions why a stakeholder engagement mechanism is needed to strengthen support for a solution.
Significant	The literature refers directly and frequently to the barrier, while its impact seems significant. For example, in the case of ‘technological’ barriers, the literature concentrates on the critical role of enabling technologies to support transitions to cleaner options.

*x*_*k,j*_ : the size of barrier type *j* for mitigation option *k* based on the literature review, which *x*_*k,j*_ ≤ 25%

*s*_*k, j*_: the size of barrier type *j* for mitigation option *k* based on the literature review *i*, which *s*_*k, j,I*_ ≤ 25%

Fourth, the study then synthesized the data from the expert survey and literature review barrier scores to come up with a final barrier score for each kind of barrier and solution. To construct that overall score, a weighted average that placed greater weight on the survey than the literature was used: the expert survey was accorded the weight of 0.7; the literature review assessment was accorded a weight of 0.3 (see equation (3)). The decision to give more weight to the survey reflected the belief expert survey offered a more accurate and recent assessment of the influence of the barrier in question. This determination was based on the fact that the questions in the survey were directly related to the issues and challenges in this study. In contrast, most of the literature was not focused on barriers but covered a broader range of sector specific technical issues. In addition, while every effort was made to use recent literature, the surveyed studies were a little more than six years old on average. However, to safeguard against criticism that the 0.3 to .0.7 ratio may skew results to the survey, a sensitivity analysis was also conducted. That sensitivity analysis assigned weight ratios of 0.6 to 0.4 and

0.8 to 0.2 to the surveys and literature reviews. The results of that sensitivity analysis are illustrated in the error bars in figure 3; the difference in weighting schemes did not significantly alter the results of the barrier analysis.

\begin{eqnarray*}{b}_{k,j}=0.7\times {\bar{x}}_{k,j}+0.3\times {x}_{k,j}\end{eqnarray*}

\begin{eqnarray*}{B}_{k}=\displaystyle \displaystyle \sum _{j}{b}_{k,j}\end{eqnarray*}

where

*b*_*k, j*_: the magnitude of barrier type *j* for mitigation option *k* combined with the expert survey and the literature review, which *b*_*k, j*_ ≤ 25%

*B*_*k*_: the total magnitude of barriers for mitigation option *k* combined with the expert survey and the literature review, which *B*_*k*_ ≤ 100%

Fifth, the study focused on how much the different types of barriers slowed diffusion of a particular solution. To make that determination, converting the composite assessment of the magnitude of the barriers into changes in how quickly a technology diffused is important. The approach to ‘slowing diffusion’ is described below.

### 4.2. Translating barriers into delays

The approach used herein involves interpreting magnitude of barriers as a delay over *a 15- year period*—that is, a ‘delay’ before an option was adopted or ‘started to diffuse.’ The assumption of a 15-year period is set as a default value. The selection of this interval is based on studies that show transitions to sustainable technologies can be between ‘1 and 16 years’ (Lund 2006, Sovacool 2016)—though some views that transitions can be lengthier contested processes (Grubler 2012). It nevertheless merits highlighting that the possible period for solutions may vary greatly across different technological and social changes and across different stages of innovation processes (Bento and Wilson 2016). Further, a possible drawback of making these assumptions is that Thailand have demonstrated at least modest levels of uptake and effectiveness for the different technologies or solutions that could influence whether the length of the assumed delay.

**Figure 4.**
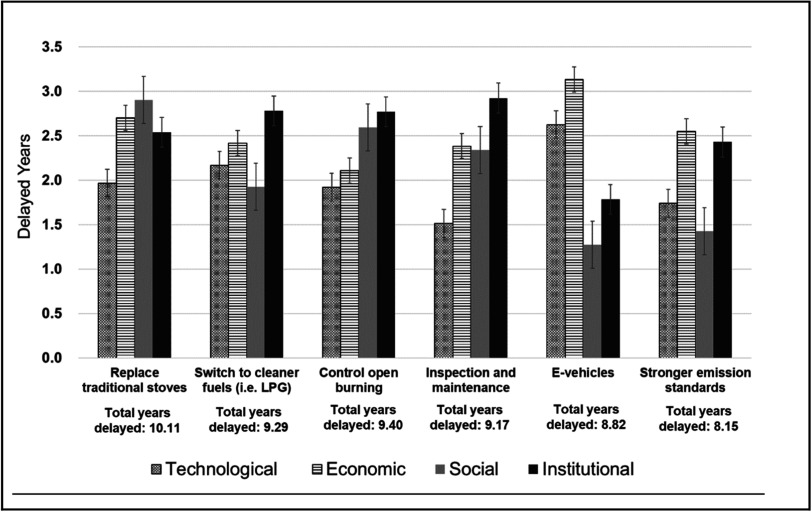
Estimated delays.

Despite these limitations, the delay in the adoption approach has the advantage of being relatively easy to interpret and incorporate into modelling scenarios. It is also likely that using an expert survey will help to account for starting points and the level of barriers at any given point in time. To illustrate both of these advantages, if the estimated reduction in diffusion rate was 22% over the assumed 15-year period, this would translate into a delay of slightly more than approximately 3 years (i.e., 0.22 × 365 days × 15 years = 1204 days = 3 year, 3 months and 19.5 days), as shown in equation (5). Taking this calculation further, if both the institutional and social barriers were slowing the diffusion, this would involve a sum of the percentages associated for each type of barrier and then multiplying that sum by the overall time period as shown in equation (6). The results of this approach are presented in figure 4 below.

\begin{eqnarray*}{t}_{k,j}=T\times {b}_{k,j}\end{eqnarray*}

\begin{eqnarray*}{t}_{k}=\displaystyle \displaystyle \sum _{j}T\times {b}_{k,j}\end{eqnarray*}

Where

*b*_*k, j*_: the magnitude of barrier type *j* for mitigation option *k* as a delay rate (reduced rate) in the speed of diffusion, which *b*_*k*, *j*_ ≤ 25%

*T*: the maximum delayed period (i.e. 15 years)

*t*_*k, j*_: the time delayed for diffusion of mitigation option *k* due to of barrier type *j*

*t*_*k*_: the total time delayed for mitigation option *k* due to barriers, which *t*_*k*_ ≤ 15 *years*

The results presented in figure 4 are illuminating for several reasons. First, the results suggest the combined impacts of the barriers are significant. In many cases, those impacts are estimated to be delays of eight or more years in total. This would be enough to undermine the prospects of achieving the full benefits of the proposed solutions. Second, the magnitude of the types of barriers vary across solutions. The technical and economic barriers are greater for the electric vehicles and emissions standards; however, the institutional and social barriers are greater than the technological and economic barriers for the other four solutions. The relatively greater magnitude of these barriers is important because these are the types of hurdles that are not explicitly factored into co-benefits modelling. Third, the lack of explicit consideration of institutional and social barriers may also lead to a discounting of the kinds of reforms needed to support the adoption and spread of a solution.

### 4.3. Applying the feasibility assessment to policies

**Figure 5.**
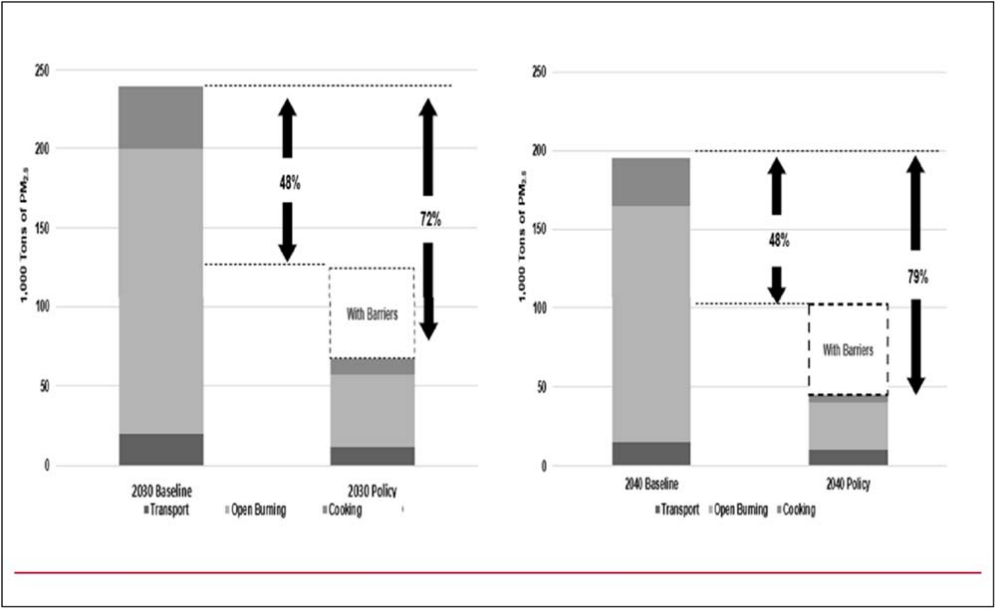
Indicative illustration of impacts from PM_2.5_ of barriers to implementing transport, cooking and open burning solution in 2030 and 2040.

One reason the results from this approach can prove illuminating is it is relatively easy to see their effects on policies. Figure 5 demonstrates those effects for policies in Thailand’s PM_2.5_ Action Plan for fine particulate emissions (the key interventions are listed in table 3).

Figure 4 depicts the emission levels for three scenarios. The first baseline scenarios demonstrate PM_2.5_ emissions without any policies. The second policy scenario demonstrates emissions of PM_2.5_ with perfect implementation of policies. The dotted box on top of the policy scenario labeled ‘with barriers’ suggests the size of the effects of the barriers on the actual reductions--that is, those amounts would be actually emitted but not be reduced due to delayed implementation considering barriers shown in figure 4.

As suggested in figure 4, the effects of the barriers are significant; the levels of emissions for all of the sectors in the with-barriers scenario is about 24% (in 2030) to 31% (in 2040) greater than would be achieved in the no-barriers effective implementation scenario. Further, the effects are most notable for open burning from agricultural residue and, to a lesser extent, clean cooking. The results underline assuming effective implementation might lead to unrealistic assumptions of what can be achieved. It also implies a need to think carefully about the enabling environment to bring estimated results in line with actual implementation conditions.

### 4.4. Overcoming implementation barriers at the policy level in Thailand

There may also be potential enabling reforms that could help overcome key barriers. The section provides an overview of proposed reforms in Thailand divided into the transport, residential energy, and agricultural sectors. More detailed descriptions of enablers are presented in table 4.

**Table 4.** Specific solutions and enabling reforms.

Sectors	Solutions	Relevant policies	Enabling reforms
Transport	Implementing Euro 5 and 6 emission standards in 2025 and 2027, respectively and fuel quality with sulphur not more than 10 ppm	*Thailand PM2.5 National Action Plan* Euro 5 Emission Standards for new vehicles	•Mechanism to coordinate with climate, air pollution, transport, energy, and industrial agencies on fuel quality improvements and tighter emission standards
		Euro 6 Emission Standards for new vehicles	•Awareness raising on the benefits of better fuel quality and tighter emission standards
		Fuel Quality with sulphur not more than 10 ppm *Thailand PM*_2.5_	•Programme to assess policy effectiveness
			•Financial support for refineries to switch to produce low sulphur fuels (reallocation of fossil fuel subsidies) and for automotive industry to switch to produce tighter emission standard vehicles.
	Promotion of EVs	*National Action Plan*	•Mechanism to coordinate with climate, air pollution, transport, energy, and industrial agencies
		Targets as follows:	•Financial support for purchasing e- vehicles
		•motorcycles 650,000	•Financial support for charging stations
		•light passenger car 440,000	•Awareness raising on the benefits of e- vehicles
		•buses 33,000	
		•trucks 34,000	
		•1,450 charging stations with 12,000 charging ports.	
	Inspection and maintenance	Gradual phase out of older vehicles	•Financial support for vehicles users to finance retrofits
Residential Energy	More efficient or cleaner stoves	Based on recent efforts from the Ministry of Energy to promote clean cookstoves, marking a return to approach used from 2008 through 2011	•Financial support for more efficient stove producers
			•Financial support for shifting to liquid petroleum gas and biogas
			•Dissemination of cleaner stoves
			•Awareness raising on the benefits of cleaner stoves
Agricultural and forest fire control	Zero agricultural residue burning	*Thailand PM2.5 National Action Plan* 90% reduction in agricultural residue burning	•Increased enforcement capacity for regulatory agencies
			•Strategic use of monitoring technologies (including satellites and low-cost sensors)
			•Incentives to purchase baling machines, mulching equipment and other management technologies
			•Creation of sustainable value chains to manage and convert residue into products
			•Awareness raising on the benefits of non-burning agricultural practices and improved air quality

For the transport sector, there is a clear need to strengthen the integration between the proposed reforms and transport, climate, air quality and health planning. This is occurring to some extent as the work on e-vehicles features in Thailand’s Nationally Determined Contribution and other climate plans, and efforts to strengthen inspection and maintenance and vehicle standards are part of the PM_2.5_ Action Plan. Strengthening institutional coordination could break siloes, boost capacities and reduce redundancies in implementing solutions. In addition, enhancing institutional coordination would also shed light on the effects of e-vehicles on air quality, while illustrating how tighter standards and inspection and maintenance on air pollution, health, and climate change. Beyond these institutional reforms, policy signals that make clear the long-term goal of transitioning to e-vehicles, commitments to financial incentives for the purchase of these vehicles, and efforts to expand charging station networks would reduce private sector and consumer uncertainties. In addition, strengthening oversight and funding for inspection and maintenance and platforms for dialogues with vehicles manufacturers and refineries on vehicles standards would be helpful. The latter set of reforms are important since proposed attempts to tighten standards can be delayed due to challenges of switching to lower sulfur fuels.

For residential energy, there is also a need to enhance integration across relevant sectoral remits and administrative portfolios. More explicit inclusion of residential energy in Nationally Determined Contributions and climate policy discussions and mechanisms facilitating planning across key divisions would help in this regard. It also may be useful to raise the profile of those issues and consider a 10-year program that works with universities and civil society partners to identify and promote alternatives that work in different contexts in Thailand. This could help build a reliable market for cleaner stoves, cleaner fuels and alternative technologies such as biodigesters and off-grid renewable applications. It would further help boost awareness levels and trigger demonstration effects that can help shift markets and mindsets on these issues. In all cases, there could also be a greater emphasis on follow-up and review and monitoring of impacts.

For the open burning of agricultural residue, there has been some recent progress due to growing political commitments and related push from top leadership. However, open burning and forest fires remain persistent problems in Thailand due, in part, to the challenges of enforcing outright bans on the practice (Kim Oanh 2013a). In this case, it might also be helpful to build stronger horizontal links across the environmental, agricultural and health agencies as well as vertical links between national and subnational governments. These links could help bring more funding to resource-constrained governments and increase investments in awareness of the impacts of alternatives such as mushroom or production of rice straw derived pellets (though depends on the mixture and composition of biomass (Kim Oanh 2013b); on- site microbial degradation or a mechanical rice straw (RS); or baling machine to collect ground biomass for off-site uses (Kanokkanjana and Bridhikitti 2007). Greater reflection on how to create markets for sustainable biomass use with circular economy models in, for example, furniture manufacturing might also gain traction.

### 4.5. Applying the feasibility assessment to carbon finance projects

One of the strengths of the approach presented in this article is that can apply to interventions at different scales. For example, a slightly modified version of the steps in section 4.1 could assess the feasibility of implementing the aforementioned climate finance cookstove project in section 2. More concretely, one could conduct a survey of users and local experts to estimate the effects of different barriers. This could then be combined with a review of field studies and other literature on the same barriers. The estimated size of the barriers could further be converted into delays over the anticipated project lifetime. Those delays could be incorporated into an assessment of emissions reductions and their associated co-benefits under ‘perfect implementation’ and ‘with barriers’ scenarios. In an additional sixth step, project developers can work with local decision makers and affected communities to determine how barriers can be overcome.

**Figure 6.**
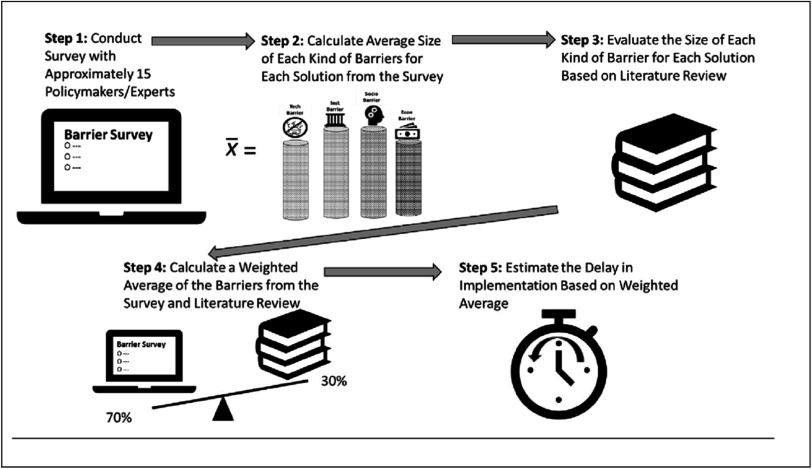
Integrating the five-step approach to estimating barriers and delays into the development of a voluntary carbon finance cookstove project *Source: Authors Diagram*.

Figure 6 illustrates how the proposed now six-step approach could be integrated into the process for the aforementioned cookstove voluntary carbon project in section 2. Note that a few modifications, underlined on the revised figure, are suggested in downscaling. These include an even greater weight on the barrier survey as relevant field studies and literature are likely to be more limited in number and scope. As also suggested in figure 6, most of the adapted project level approach is likely fit in part 4 on sensitization of a typical voluntary carbon finance project development process; an additional step might be checking the estimated delays against actual part 7 on implementation.

Applying the feasibility assessment on smaller scale could be useful for a few reasons. Most notably, examining barriers for a community level project is likely to offer a context-rich understanding of how much issues such as social acceptance influence implementation. This is particularly important because social acceptance is likely to vary not only across a country but communities. Applying the approach at the project level might also facilitate closer communication with affected stakeholders and project developers. This could, in turn, boost ownership of enabling reforms such as awareness raising programmes. Finally, efforts to downscale could also complement tools that have been used for analyzing factors influencing acceptance of new practices in areas such as agriculture (Kuehne *et al* 2017).

While there are advantages of downscaling from national policies to local projects, obtaining survey responses and locating relevant literature may take time and resources. For instance, administering a survey to those with sufficient expertise to comment on multiple dimensions of feasibility for a specific project may require a strong local network and investing in awareness raising activities. Yet, it may be possible to reduce that time and resources by mainstreaming the approach into existing sensitization materials. It may also be possible to integrate these efforts to longstanding efforts to bring more systematic assessments of sustainable development benefits and the feasibility of achieving them into carbon finance mechanisms. Discussions are ongoing of using a more systematic assessment of SDG benefits to evaluate activities funded under Article 6.4 of the Paris Agreement (UNFCCC 2023).

## 5. Conclusion

This study has argued multiple dimensions of feasibility deserve more attention in research on co-benefits. It then developed a novel approach for incorporating different dimensions of feasibility into co-benefit modelling. Making these links is useful because integrated assessment models often assume different packages of policies correspond with different emission pathways. The framework presented herein suggests that movement along emissions pathways depends on the package of policies and reforms enabling their implementation. Reflecting on enabling reforms should therefore receive more attention in the modelling of how policies affect emissions. This added attention is particularly important for actions that require enabling support to drive behavioural and social changes.

While this study’s main results therefore can help refine co-benefits modeling, they also raise questions about the broader application of its findings. This section outlines similar methods to those in this study could be extended to other contexts.

One extension would be conducting a barriers analysis in other countries. Building a barriers database that contributes to a more general feasibility framework could help generate average lengths of implementation delays. It would then be possible to extrapolate results to countries with similar conditions and validate the results with stakeholder consultations. This could also be complemented by updated literature reviews and weighting schemes. Making sure assumptions and methods used to make these assessments and weighting are transparent will be critical.

Another extension involves making the link between the feasibility assessment and work on technological diffusion. Studies of different diffusion functions for innovation have made significant headway in modelling factors that impact the spread of solutions. For instance, the Bass diffusion model (Bass 1969, Norton and Bass 1987, Brown 2011, Radomes and Arango 2015) is a model that presents how two factors—innovation and imitation—influence technological diffusion. This work could be more explicitly linked to modelling scenarios. There are indeed already extensions of the Bass model that bring integrate technology prices from government policies that suggest potential synergies with the approach demonstrated in the paper (Bitencourt *et al* 2021).

A related extension involves connecting the feasibility assessment to the costs of enabling reforms. In this case, there is sizable literature on transaction costs that can offer insights into the costs of creating and running public programs under different institutional conditions (Falconer 2000, Falconer and Saunders 2002, Vatn *et al* 2001, Rorstad *et al* 2007, Kuperan *et al* 2008, Mettepenningen *et al* 2009). This kind of cost information could be fed into modelling frameworks to understand the resources needed to lower non-economic and non- technological barriers. It could also offer an interesting complement to some of the work that has focused on how enabling policies such as subsidies or carbon taxes alter selections of different technologies.

Another extension involves systemic changes that can overcome barriers. In this case, though barriers appear significant for some of the solutions, changing within and across socioeconomic systems may help break them down. For example, the transition to cleaner stoves and fuels may move forward as with more market access for cleaner options and lower energy prices. The proposed transition may nonetheless be difficult due to resistance from existing resource-intensive systems. One way to drive systemic change is to identify leverage points with the greatest potential at different scales. In fact, one of the core insights of work transitions is that complementing larger landscape changes with small scale niches can lead the way to systemic changes. Future studies could use surveys to identify where it is feasible to complement small-scale cooking projects with institutional reforms that support air quality plans to drive transformative change.

## Data Availability

All data that support the findings of this study are included within the article (and any supplementary files).

## Appendix. List of survey respondents and surveyed literature cookstoves

**Table:**

Country	Profession	Affiliation
Thailand	Researcher/academic	Asian Institute ofTechnology
Thailand	NGO	Center for Creativity andSustainability
Thailand	Researcher/academic	Lampang Rajabhat University
Thailand	Researcher/academic	Ubon Ratchathani University
Thailand	Researcher/academic	Center for Clean Air Solutions
Thailand	Lecturer	Pibulsongkram Rajabhat University
Thailand	Researcher/academic	Srinakharinwirot University
Thailand	Researcher/academic	Science & Industrial Tech. PSUSurat
Thailand	Government official/policymaker	Department of Alternative Energy Development and Efficiency
Thailand	Government official/policymaker	Department of Alternative Energy Development and Efficiency
Thailand	Researcher/academic	King Mongkut’s University of Technology North Bangkok
Thailand	Researcher/academic	Royal Forest Department, BKK
Thailand	Governmentofficial/policymaker	Department of Alternative Energy
Thailand	Private sector/business	Ratchaburi province
Thailand	Governmentofficial/policymaker	National Research Council ofThailand
Thailand	Private sector/business	Representative of the community enterprise group, manufacturer and distributor of high efficiencycharcoal cooking stoves
Thailand	Governmentofficial/policymaker	Provincial energy office of ChiangMai.
Thailand	teacher and entrepreneur	Chiang Mai Rajabhat University/Lanna Technology
Thailand	Governmentofficial/policymaker	Royal Forest Department
Thailand	Researcher/academic	Expert center of inovative clean energy and environment TISTR
Vietnam	NGO	Vietnam Clean Air Parnership(VCAP)
Cambodia	Governmentofficial/policymaker	Phnom Penh, Cambodia
Philippines	NGO	Clean Air Asia
Germany	Private sector/business	GIZ
Vietnam	Project developer	SNV Vietnam
Vietnam	Governmentofficial/policymaker	Hanoi Environmental ProtectionAgency
Singapore	Governmentofficial/policymaker	National Environment AgencySingapore

### 5.1. Open burning

**Table:**

Country	Profession	Affiliation
Cambodia	Researcher/academic	Royal University of Phnom Penh
Indonesia	Researcher/academic	Itenas Bandung Indonesia
Malaysia	Researcher/academic	Universiti Kebangsaan Malaysia
Myanmar	Private sector/business	Environmnetal Quality Management Co., Ltd, Yangon, Myanmar
NA	Retired Government Official	Before my retirement I worked at the Environmental Research and Training Center, Department of EnvironmentalQuality Promotion
Philippine	Researcher/academic	Manila Observatory and Ateneo deManila University (Department of Physics)
Philippine	Researcher/academic	Manila Observatory
Philippine	NGO	Clean Air Asia
Philippine	Researcher/academic	Clean Air Asia
Singapore	Researcher/academic	University
Thailand	Government official/policymaker	Air Quality and Noise ManagementBureau, Pollution Control Department, Bangkok, Thailand
Thailand	Researcher/academic	King Mongkut’s Institute of TechnologyLadkrabang
Thailand	Researcher/academic	National Astronomical Research Instituteof Thailand (NARIT)
Thailand	Researcher/academic	Thammasat University
Thailand	Researcher/academic	Thammasat University
Thailand	Researcher/academic	Asian Institute of Technology
Thailand	Government official/policymaker	Pollution Control Department, Ministry ofNatural Resources and Environment
Thailand	Researcher/academic	Valaya Alongkorn Rajabhat Universityunder the Royal Patronage
Thailand	Researcher/academic	King Mongkut’s University of TechnologyThonburi (KMUTT)
Thailand	Researcher/academic	Chiang Mai University
Thailand	Researcher/academic	Department of Agronomy, Faculty ofAgriculture, Kasetsart University
Thailand	Researcher/academic	Chiang Mai University
Thailand	Researcher/academic	Asian Institute of Technology, Thailand
Thailand	Researcher/academic	Faculty of Public Health, ThammasatUniversity, Thailand
Thailand	Researcher/academic	Asian Institute of Technology
Vietnam	Researcher/academic	Institute for Agricultural Environment, HaNoi, Viet Nam
Vietnam	Researcher/academic	Hanoi University of Science andTechnology, Hanoi, Vietnam
Vietnam	Researcher/academic	Hanoi, Vietnam
Vietnam	Researcher/academic	Vietnam Clean Air Parnership (VCAP)
Vietnam	Researcher/academic	Faculty of Environment, University of Science, VNUHCM, VietnamNational University Ho Chi Minh City

### 5.2. Transport

**Table:**

Country	Profession	Affiliation
Myanmar	Researcher/academic	Private sector
Thailand	Researcher/academic	King Mongkut’s Institute of Technology Ladkrabang
Thailand	Researcher/academic	Siam University, Bangkok
Thailand	Researcher/academic	KMUTT
NA	Researcher/academic	University
NA	Government official/policymaker	Environmentalist
Thailand	Researcher/academic	King Mongkut’s University of Technology Thonburi (KMUTT)
Thailand	Researcher/academic	Ayutthaya, Thailand
NA	Government official/policymaker	Department of Land Transport
Thailand	Researcher/academic	Transportation Institute, Chulalongkorn University
Thailand	Researcher/academic	King Mongkut’s University of Technology Thonburi
NA	Private sector/business	Grutter Consulting
Thailand	Researcher/academic	Asian Institute of Technology
Thailand	Researcher/academic	Chulalongkorn University
Vietnam	Researcher/academic	Hanoi University of Science and Technology, Hanoi, Vietnam
Indonesia	Researcher/academic	Itenas Bandung
Thailand	Researcher/academic	The Joint Graduate School of Energy and Environment
NA	Researcher/academic	KU
Thailand	Researcher/academic	ENTEC NSTDA
Thailand	Researcher/academic	Thammasat University
Thailand	Researcher/academic	Lecturer
Thailand	Government official/policymaker	TISI
Thailand	Private sector/business	Thailand Ayutthaya, HATC-MA
Thailand	Researcher/academic	Center for Clean Air Solutions
Thailand	Government official/policymaker	Pollution Control Department (Thailand)
Thailand	Environmental Consultant	United Nations Industrial Development Organization
Thailand	Researcher/academic	PTT Public Company Limited
Thailand	Researcher/academic	Asian Institute of Technology, Thailand
Thailand	Researcher/academic	Faculty of Public Health, Thammasat University
Thailand	Development organization	UNEP
Thailand	Researcher/academic	Asian Institute of Technology
Thailand	Engineer	Honda Automobile(Thailand)
Thailand	Government official/policymaker	Thailand
Germany	International cooperation company	GIZ
NA	Government official/policymaker	Department of Land Transport
Australia	Researcher/academic	Australian Embassy Bangkok
Thailand	Researcher/academic	Department of Environment, Air Quality and Noise Management Division
Thailand	Researcher/academic	Nakhon Ratchasima, Thailand

**Table:**

No barrier/insignificant information	+	Small	++	Moderate	+++	Significant
Source	Location/Years	Summary	Tech	Econ	Social	Inst
Li and Crawford-Brown 2011	Bangkok Metropolitan Area, 2000–2010	This study finds that the benefits clearly outweigh the costs of an I/M programme in Bangkok.		+		
Li 2017	Bangkok Metropolitan Area, 2010–2015	This study argues that the design and the implementation of I/M programme influences their effectiveness.				+++
Dandapat *et al* 2020	Kolkata and Howrah, India, 2015–2020	This study assesses the performance of I/M programme in Kolkata and Howrah, India.				+++
Kholod and Evans 2016	Russia, 2010–2015 (note that this applies to all diesel emissions)	This study assesses Russia’s approach to controlling diesel emissions by looking at of I/M programmes, noting the lack of training and the inspecting center, perverse incentives in programme design, and limited government oversight.				+++
Baptista Ventura *et al* 2020	Brazil, 2015–2020	This study evaluates the impacts of a poorly implemented I/M programme as well as the need for improvements in public transport in Rio De Janeiro, Brazil.	++			+++
Tungsuwan *et al* 2021	Thailand, 2020–2030	The article focuses on the policies that Thailand has adopted to promote e-vehicles, raising questions about the policies and supportive technologies that needed by 2030 to create a market for e-vehicles.	++	++		++
Thananusak *et al* 2017	Thailand, 2020–2030	This study examines factors affecting acceptance of e-vehicles in Thailand, contending consumer perceptions of performance of the e-vehicle is critical.	+++		+++	
Vongurai 2020	Thailand, 2020–2030	This study evaluates factors affecting willingness to purchase e-vehicles in Thailand, suggesting a need for greater awareness of environmental benefits and technological advances (i.e. fuel efficiency).	++		++	
Bakker 2018	ASEAN, 2020–2030	This study employs the Avoid-Shift-Improve framework to organize policies in ASEAN countries, concluding that enforcement is often lacking.				+++
The National 2020	Thailand, 2020–2030	This study examines stringent emissions standards in Thailand and complaints from the electric vehicle industry.				+++
Subramanian *et al* 2009	Thailand, 2010–2020	This study employs a technical/atmospheric analysis of the Developing Integrated Emissions Strategies for Existing Land Transport (DIESEL) project, finding that emissions reductions in PM_2.5_ are significant only for heavy not light duty vehicles for tighter Euro standards on PM emission rates.	++			
Cheewaphongphan *et al* 2020	Thailand, 2020–2050	This study assesses the mitigation potential for the transport sector in Thailand, underlining delays in the tightening of emission standards and that institutional and policy factors are responsible for the delays.				+++
Zhang *et al* 2018	Global, 2005–2100	The study uses integrated assessment/computable general equilibrium model to illuminate relationship between the transport sector and the macroeconomy, noting that technological transformations in the transport sector are possible but require stronger linkages with climate/development policies.	++	++		
Sharma and Jain 2019	India	This study concentrates on the costs of household cooking fuels and impediments slowing the choice of clean household energy in India’s Indo-Gangetic India.	++	++	+++	+++
Vigolo *et al* 2018	Global	This article suggests that more than access/affordability are needed to promote improved cookstoves ICS.	++	+++	+++	+++
Thoday *et al* 2018	Indonesia	This study concentrates the impressive transition in the Zero-Kero program in Indonesia.	++	+++	++	+++
Dendup and Arimura 2019	Bhutan	This study concentrates on how the provision of information facilitating the uptake of clean cooking fuel in Bhutan—with some variation depending on education levels etc	++	+++	++	+++
Chalise *et al* 2018	India	The study concentrates on the challenges to biogas and other clean cooking interventions in India, noting that the accumulation of technical knowledge helps to curb resistance to clean cooking interventions.	+++	++	+++	++
Kshirsagar and Kalamkar 2014	Global	This study reviews literature on barriers to improved cookstove programmes, underlining the key role of institutional infrastructure.				+++
Kanokkanjana and Garivait 2013	Thailand	This study calls for alternative straw management practices in Thailand in an estimation of carbon content loss from burning of rice straw.		+++	+++	
Pearson *et al* 2016	Global	This report focuses on opportunities and challenges to curbing open burning in the Himalayas with an emphasis on India, it also suggests that cultural factors and lack of awareness of soil properties make resolving the problem difficult.		+++	+++	
Bhuvaneshwari *et al* 2019	India	This study concentrates on difficulties with limiting burning in India, and underlines a lack of institutional mechanism to manage crop residue is a major hurdle.		+++	+++	+++
